# Carlos Asensio and the dawn of molecular microbial ecology

**DOI:** 10.1007/s10123-024-00596-6

**Published:** 2024-10-03

**Authors:** Víctor de Lorenzo, Fernando Baquero, Alfredo Aguilar

**Affiliations:** 1 Departamento de Biología de Sistemas, Centro Nacional de Biotecnología, CSIC C/Darwin, 3 Madrid-Cantoblanco, 28049 Madrid, Spain; 2 Departamento de Microbiología, Instituto Ramón y Cajal de Investigaciones Sanitarias (IRYCIS), Hospital Universitario Ramón y Cajal, 28034 Madrid, Spain; 3 Directorate-General Research and Innovation (Ret.), European Commission 1049, Brussels, Belgium

**Keywords:** Carlos Asensio, Microcins, Microbial and molecular ecology, Microbiome, Science policy

## Abstract

At near 50 years of the discovery of microcins, this article highlights the pivotal—but under-recognised—influence of Spanish biochemist Carlos Asensio (1925–1982) in contemporary microbiology, featuring the epistemological, sociological, and cultural impact of his scientific achievements. At a time when the intestinal microbiome is central to current biomedical research, it is due to emphasise his role in the establishment of new scientific fields that are now considered fundamental. Despite his premature death at the peak of his conceptual and experimental creativity, many of his ideas about microbial communication in complex communities inspired a generation of researchers and opened new topics reach to this day. Asensio was also a trailblazer in Spain, advocating for fundamental research within the socio-economic context of his time. He foresaw the shift towards what is now termed the knowledge-based bioeconomy, recognised the need for multidisciplinary research teams, and advocated integration science into societal and political agendas. These facets became evident during his research on microcins, low molecular weight bioactive compounds produced by enterobacteria. These molecules were hypothesised as mediators of microbial interactions in the human gut and were considered potential new antibiotics and even antitumoral agents. His research mobilised young talent and attracted unprecedented resources in Spain during the late 1970s–early 1980s. It underscored the medical value of microbial ecology and exemplified the benefits of collaboration between academia and industry. Asensio played a pivotal role in the emergence of molecular microbial ecology as a research discipline and its foundational and applied significance in biotechnology.

## Introduction

Biomedical research over the past decade has revealed a new and unexpected set of factors that determine both individual and population health based on their material conditions and lifestyles. It is not surprising that these factors have become rational intervention targets, either through new therapies and treatments or through measures impacting population health management. Examples include the growing challenge of antibiotic resistance, or the effects of climate change and the environmental microbiome on public health. Among these new factors, the growing evidence of the influence of the intestinal microbiome on virtually all aspects of human body function stands out (Gomaa 2020).

It has long been known that the digestive tract is a dynamic ecological niche populated by a large number of bacterial species, which can constitute up to 30% of the weight of faeces. In fact, the number of bacteria in our intestinal tract equals, and at times exceeds, the number of eukaryotic cells in the body (Sender et al. 2016). It has also been a common assertion in microbiology texts that the bacterial population in the gut provides essential vitamins for human metabolism. However, it is only with the development of massive DNA sequencing methods and *omics* technologies (proteomics, metabolomics, transcriptomics, among others), which enable molecular studies independent of traditional microbial culture, that awareness has arisen of the intimate interaction between microorganisms inhabiting our body and practically all biological functions recognised as typically human. On one hand, metagenomics (i.e., sequencing and analysis of the entire genetic complement of a niche based on its total DNA contents) has revealed the immense diversity of prokaryotes (bacteria, viruses, and archaea) colonising the intestine, their distribution along its various segments, their variations with diet, age, and lifestyles, and even their correlation with diseases whose aetiology had previously been attributed to other causes (Pflughoeft and Versalovic 2012). On the other hand, more recent studies have unveiled intensive chemical signalling traffic between the intestinal microbiome and neuronal functions in what has been termed the *gut-brain axis* (Mayer et al. 2015), influencing or at least affecting moods, sociability, and even associating with psychiatric conditions (Appleton 2018). Indeed, the intestinal microbiome is now considered a *third brain* of the human body, alongside the neuronal and immune systems, capable of processing environmental signals and reacting to them with specific responses. These are often elicited by small molecules that may function as neurotransmitters. Note also that the personal microbiome is not limited to isolated individuals. On the contrary, bacteria constantly flow between individuals and between them and the environment, including animals (Kates et al. 2020), plants, and surfaces of objects we come into contact in daily life. The microbiome acts as *Ariadne*’*s thread* connecting and communicating us with the rest of the biological and inert world (Tu et al. 2020) through mechanisms of chemical signal exchanges that are only now beginning to be understood (Holmes et al. 2011).

With all this new knowledge about our interaction with microorganisms, it is not surprising that studies on the microbiome and the possibility of rational exploitation and modification for preventive or therapeutic purposes are among the current frontiers in health research. The so-called faecal transplants (Kaakoush 2020), already a reality in many hospitals, are just the beginning of what many predict as a new branch of biomedicine based on new foundations and immense therapeutic opportunities.

## The quest for the missing signals in microbial ecology

**Fig. 1:**
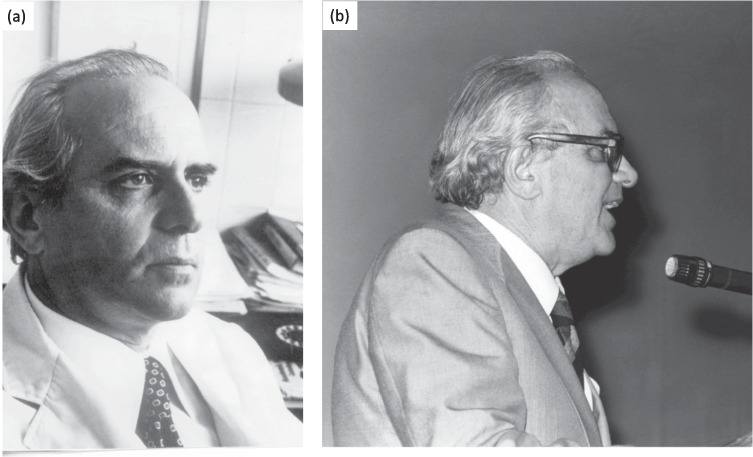
Carlos Asensio (1925–1982). **a** Family portrait (kindly provided by Lucía Asensio). **b** One of his last pictures during a conference in the Juan March Foundation (Madrid) on the New Biology a just few months before the accident that finished his life (Photo credit: Fundación Juan March, https://shorturl.at/2QFEk)

**Fig. 2:**
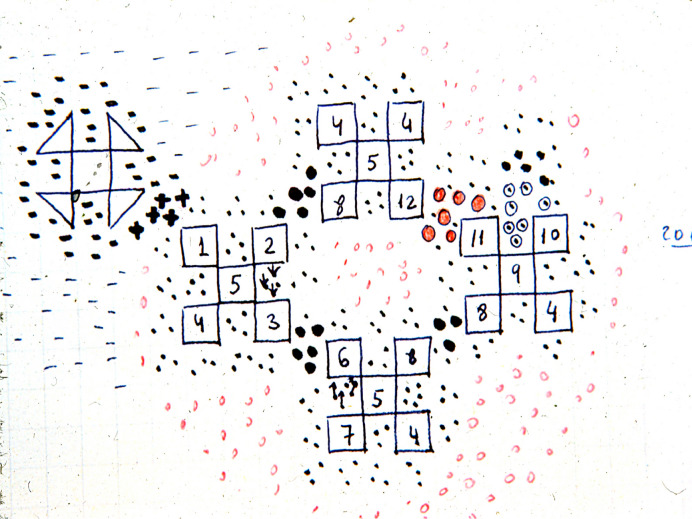
Working notes between the Asensio and Baquero teams on Microbial Ecology. Carlos Asensio and Fernando Baquero, during their results discussion sessions, shared a fondness for using graph paper to *sketch* hypotheses. Here, sets of bacterial species or clones (numbered; note organism “4” appearing in multiple sets) are depicted with dots, small circles, and dashes in black and red (all very improvised on the fly), illustrating the diversity of molecules constituting the *molecular ecology* of the microbiota (called then intestinal flora). These interactions lead to synergistic or antagonistic effects (microcins) both between bacteria and among bacterial communities (image kindly provided by F. Baquero)

In the early 1970s, in a country like Spain, at that time on the periphery of the global scientific landscape, addressing the aforementioned issues not only had to wait several decades but they were alien to the limited scientific activities and interests of those years. However, even in challenging times, talent and creativity can emerge and flourish. In this case, it happened when individuals from quite different fields crossed paths (as has often occurred in the history of research). On one hand, the young microbiologist Fernando Baquero had been documenting successions of bacterial strains in the intestines of neonates from his microbiology service at La Paz Children’s Hospital in Madrid. These strains colonised the intestines of infants who had initially sterile intestinal tracts, and their identity and diversity could be tracked in some cases (for instance, strains of *Escherichia coli*) using classical serotyping technologies. These investigations, which formed the basis of Baquero’s PhD thesis (Baquero 1973), grabbed the attention of Carlos Asensio (Fig. 1), a biochemist of the Institute of Enzymology belonging to Spanish National Research Council (acronym CSIC in its original language). From his side, Asensio had previously identified low molecular weight (LMW) molecules, as the one later identified as L-valine, that inhibited the growth of *E. coli* K12. Upon learning about Baquero’s work, both agreed to collaborate to investigate what sort of LMW compounds could mediate interactions among the strains colonising the intestine and their successions. A very intense and vibrant collaboration was then established between Asensio’s and Baquero’s teams (Fig. 2), which continued for years until the passing of the former in a tragic accident in 1982. Based on this collaboration, a set of hypotheses was formulated that, in retrospect, not only anticipated now well-known phenomena and mechanisms but also led to the discovery of a type of biomolecules whose medical interest and potential continue to the present day.

The logical process followed by Asensio, based on Baquero’s data, can be summarised as follows. First, successions of strains during intestinal colonisation and all their derivatives should originate from secretion into the environment by newcomers of molecules with inhibitory activity against preceding strains and with a broad spectrum of action. Second, these molecules should be small to facilitate diffusion in the intestinal environment and, contrary to colicins, should be resistant to proteases to survive the known abundance of these enzymes in the digestive tract. Third, these compounds should be produced in a nutrient-limited scenario, as is typical of the last section of the intestine and would likely be in a low-growth or stationary phase. He then deduced that *there must be* small molecules with antibiotic activity produced by intestinal bacteria isolated from neonates at post-birth and subsequent colonisation times. What we might call the principle of the *there must be* (Asensio 1976, 1978) is an application to ecology of the idea of the missing link that has been so useful in other fields (see below).

## The discovery of microcins

**Fig. 3:**
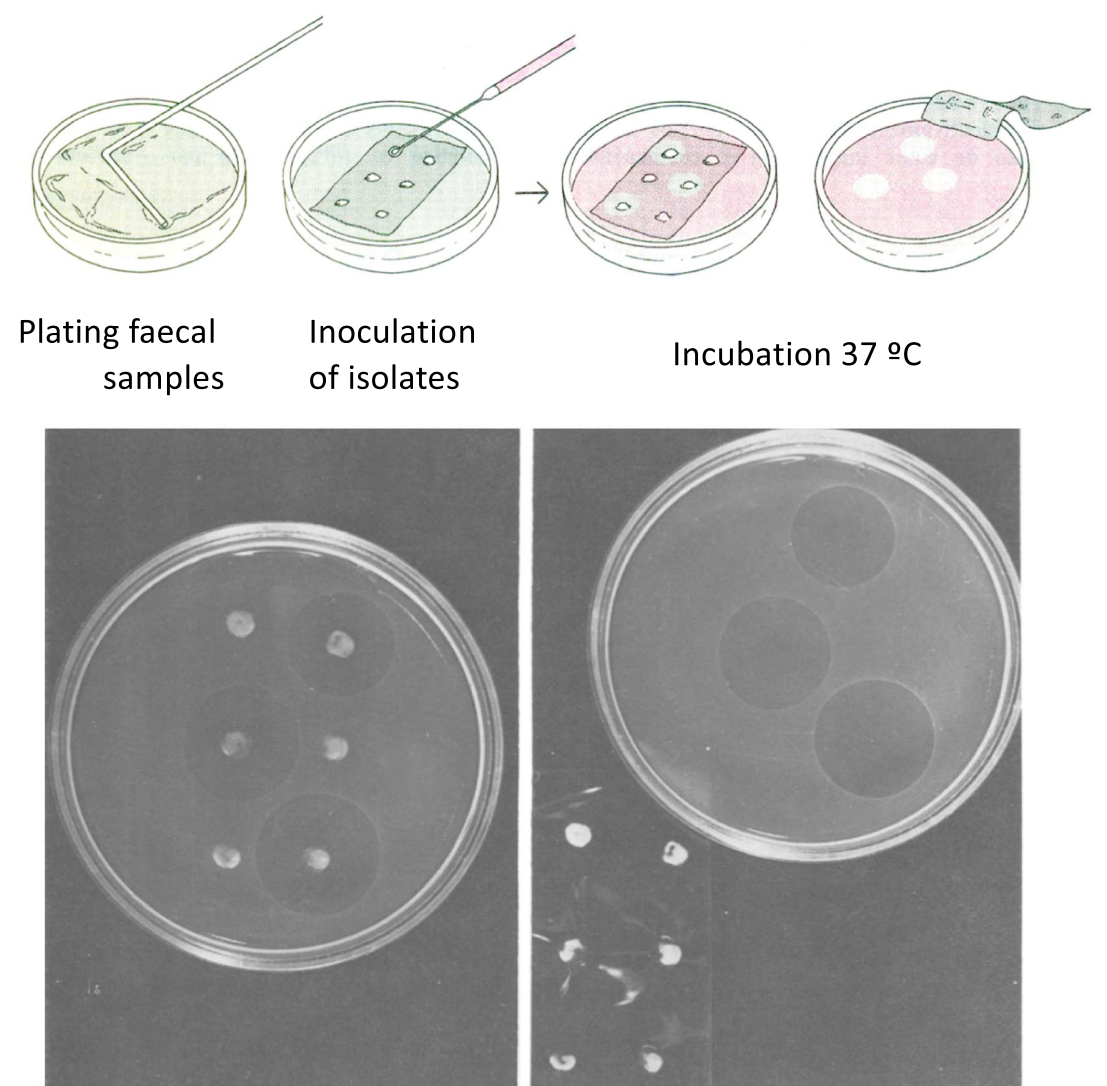
Microcins detection assay. Dense inocula (spots) of bacterial colonies, mainly derived from intestinal samples of hundreds of newborns, are inoculated onto the surface of a cellophane membrane (later dialysis tubing) placed on a minimal Davis agar plate pre-inoculated with a lawn of a bacterium that reveals antimicrobial activity (*Escherichia coli* K12). One theoretical criterion of the assay was to place a heavily loaded bacterial spot on the cellophane, so that cells presumed to produce amensalistic products would be in a very low growth or near stationary phase, similar to conditions in the colon. It was later observed that many microcins are indeed produced preferentially in stationary phase. After 24–48 h of incubation, inhibition of growth of the indicator *E. coli* by substances capable of crossing the membrane (with a molecular weight below 10,000) is observed (image kindly provided by F. Baquero)

**Fig. 4:**
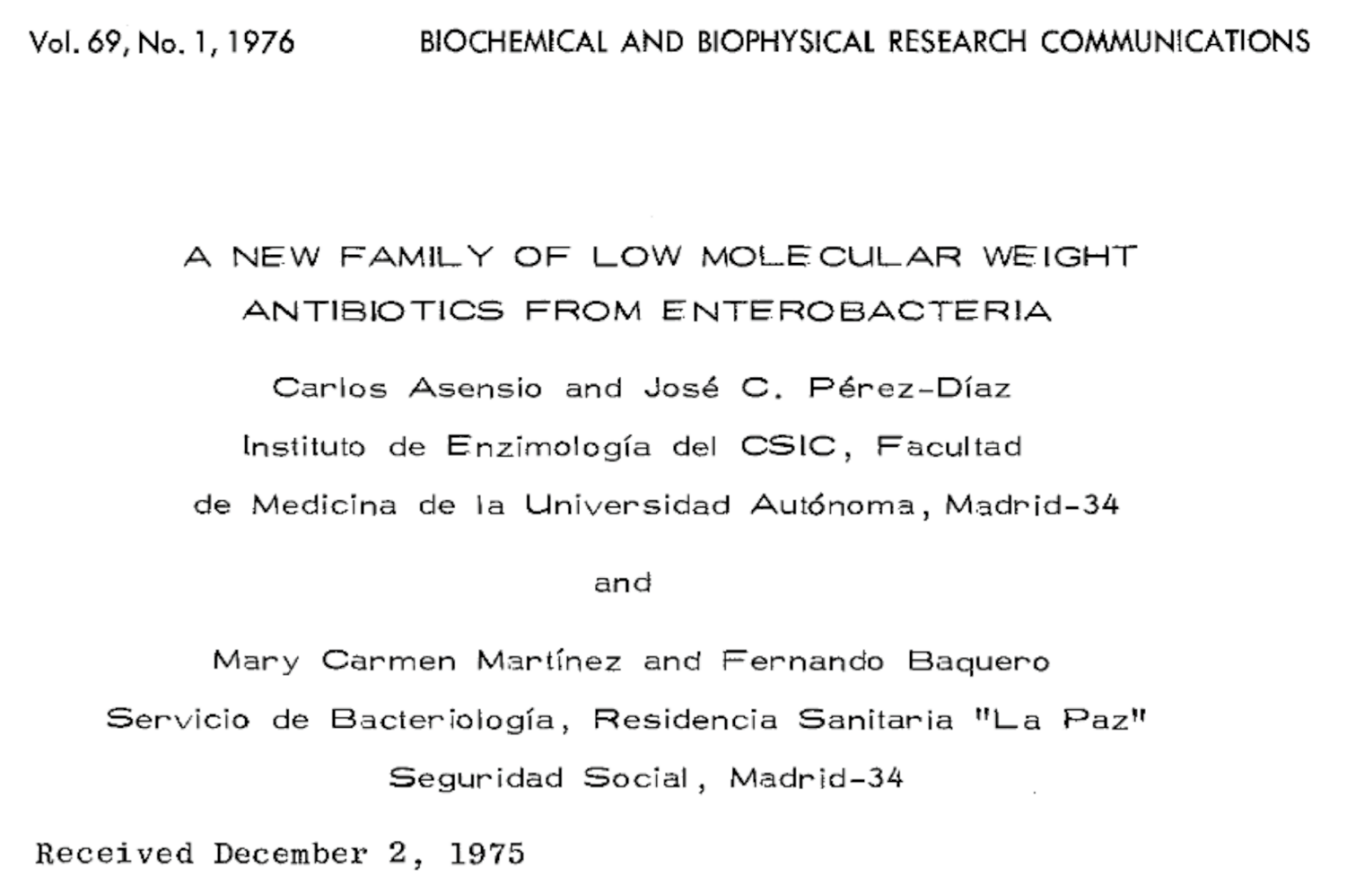
Original publication of the first paper describing the discovery of microcins. The article (Asensio et al. 1976) was manually typewritten and published as such in the (by then) rapid publication journal BBRC, which was by that time edited by the earlier Asensio’s postdoc mentor Bernard Horecker at the University College of Medicine in New York

Once the existence of low molecular weight antibiotic molecules produced in minimal media by intestinal bacteria from neonates was predicted, the next question was to design an experimental system to detect them. Here came another display of synergic creativity by Asensio and Baquero and an example of what is now termed *frugal technology* or *frugal science.* This involves a conceptually sophisticated yet extremely simple platform to implement in the laboratory. The assay in question (Fig. 3) involved seeding a homogeneous layer of a sensitive indicator bacterium (*E. coli* K12) on a Petri dish with minimal medium. A sterile cellophane membrane, later replaced by dialysis membrane (which allows only small molecules to pass), was placed on the resulting surface. On top of this, the candidate intestinal isolate, potentially producing an antibiotic, was punctually inoculated. After incubating the plate, the production of hypothesised molecules could easily be detected by the appearance of growth inhibition halos. The experiment was conducted successfully, and many enterobacteria isolated from neonates (and subsequently many other strains of intestinal origin) were identified as producers of low molecular weight antimicrobial substances. The discovery of these substances was published in 1976 (Asensio et al. 1976; Fig. 4). The molecules in question, which faithfully matched the conjecture, were termed *microcins.* The word *microcina* appeared also in Spanish in 1977 (Baquero and Asensio 1977), followed the same year by the first PhD thesis with the same title (Pérez-Díaz 1977).

A pivotal advance that sparked interest in these molecules beyond Spanish borders was Baquero’s discovery, while working at the Pasteur Institute in Paris, that the biosynthesis of some microcins was encoded on conjugative plasmids (Baquero et al. 1978). Following these developments, there was soon an initial classification of microcins into activity-immunity groups (Sánchez 1981) and the first data on their genetics—an aspect that Felipe Moreno extensively developed later at the Ramón y Cajal Hospital. Although determination of their molecular structures came much later, techniques of the time allowed for the purification of some of these molecular species and the elucidation of their mechanisms of action and resistance. Meanwhile, the Spanish-speaking public interested in scientific innovations could read an article on microcins in the magazine *Investigación y Ciencia* (the Spanish version of *Scientific American*; Asensio and Baquero 1979).

**Fig. 5:**
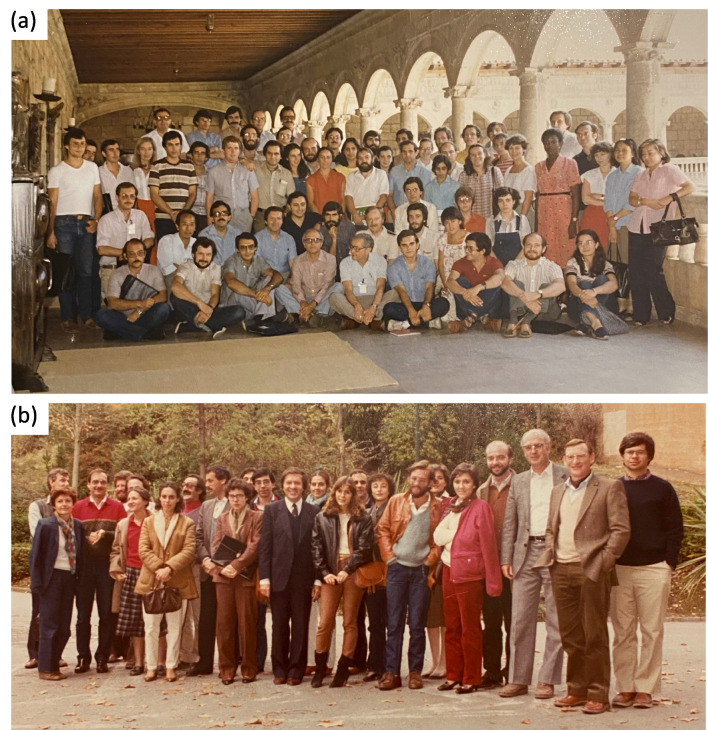
Foundational international meetings on the topic of microcins. **a** Workshop in León, July 1981 on Molecular Genetics of Antibiotics, featuring leading global specialists of the time on antimicrobial compounds. Carlos Asensio, in the front row, is flanked by Fernando Baquero and Arnold Demain. In other positions, notable researchers such as David Vázquez, Felipe Moreno, Juan Francisco Martín, Jordi Barbé, Keith Chater, and David Hopwood, can be seen. This meeting marked the first international showcase of microcins as a new class of antibiotics. **b** Meeting in Granada in December 1983, among world-renowned figures working on colicins (including J. Konisky, V. Braun, D. Cavard, M. Schwartz, R. Kolter, T. Pugsley, and C. and A. Lazdunski), and the groups of Carlos Asensio (sadly already absent) and Fernando Baquero-Felipe Moreno. At this meeting, it was decided that microcins constituted a group of microbial molecules distinct from conventional colicins, marking their international recognition. The meeting served as a tribute to the legacy of Carlos Asensio. (photos kindly provided by F. Baquero)

However, the consolidation and recognition of microcins as a new, fundamental microbiological issue with potential applications only came later, aided by two important international meetings in the early 1980s. The first one held in León in 1981, entitled *Molecular Genetics of Antibiotics,* presented microcins to an international constellation of antibiotic researchers, including prominent figures in the field such as A. Demain, D. Hopwood, K. Chater along with local experts D. Vázquez and J. F. Martín (Fig. 5a). A noteworthy anecdote of Asensio’s talk in that meeting was his reference to the Isaiah 45:8 text written in S. Waksman’s (the discoverer of streptomycin) gravestone “…*the earth shall open and bring forth salvation..*”, referring to antibiotics produced by soil microorganisms, and his proposal of the alternative motto “*…salvation shall come from yourself…*” in reference to the potential healing power of microcins from the human intestine. A second high-level meeting took place in Granada, organised by Baquero in 1983 (already without Asensio), with leading bacteriocin specialists (Fig. 5b), aimed at clearly distinguishing between bacteriocins and microcins. These two meetings and the results presented therein created enormous anticipation in the scientific community regarding the potential of these molecules to constitute a new type of antibiotics isolated not from soil microorganisms—like most antimicrobials—but from strains originating from the human body.

Apart from their inhibitory capacity, Asensio and Baquero also highlighted the possible ecological significance of microcins as the basis for what would be called *microbiotherapy*, i.e. a selective intervention against a particular pathogen based on artificial bacterial flora (Malard et al. 2021) in the context of what is now called *microbiome engineering* (Cubillos-Ruiz et al. 2021). All these developments attracted the attention of a considerable group of young Spanish chemists, biologists, and pharmacists, and shortly thereafter, Latin Americans, who saw in this topic a novel scientific project, genuinely born in Spain, with numerous fundamental scientific questions and immense translational potential. Those years can be considered the foundational moment of an entire generation of high-calibre microbiologists trained in the epistemology and scientific school of Asensio and Baquero.

But that is not all. The identification of a chemical language that modulates the composition and dynamics of populations, in this case microbial, through negative interactions added a new perspective to traditional ecology, based primarily on predator–prey cycles, competition for territory, and nutrient access. The addition of these less visible yet equally or more effective interactions, mediated by small molecules, contributed to the birth of what is now called *microbial molecular ecology*, where interactions analogous to those known and described at the macroscopic level are documented at the cellular and even subcellular level. Notably, a current branch of so-called *sociomicrobiology* uses bacterial experimental systems to assess ecological hypotheses that are impossible to verify otherwise (Harrington and Sanchez 2014; Oliveira et al. 2014). Furthermore, chemical signalling between microbiome microorganisms and these with the human host is today one of the most interesting fields of research in the current landscape (Li et al. 2013; Thaiss et al. 2016) which ultimately can trace its origins back to the seminal ideas and experiments previously mentioned.

The field of microcins has had enormous international scientific impact (more than 1000 papers recorded in PubMed at this time), which continues to grow over the years, in areas of interbacterial communication, antibiotic resistance, new structures of antimicrobial peptides (including lasso peptides; Baquero et al. 2024), antibacterial compounds, antitumor compounds, and neuropeptides.

## Carlos Asensio and his circumstances

The key figure in the developments mentioned above is the repeatedly cited Carlos Asensio. The recollection of his significant conceptual and experimental contributions to the discovery of microcins provides us with an opportunity not only to acknowledge his often-overlooked role in the Spanish scientific landscape of his time. But also, to propose him as a worthy archetype of a researcher deeply engaged with the historical context in which he lived. Today, it is impossible to comprehend the scientific process without recognising the close interplay between science, innovation, technological development, application and exploitation of results, collaboration between academia and industry, intra- and international scientific collaboration, the social impact of science, and the existence of robust scientific and industrial policies. However, in the 1960s and 1970s, none of these concepts was prevalent or even anticipated in Spain. It was during this period that Asensio, alongside other contemporary figures such as A. Sols, F. Mayor Zaragoza, M. Salas, and J. Rodríguez-Villanueva, managed to establish laboratories of excellence despite the complex sociopolitical landscape and the severely limited resources for biomedical research in Spain (Aguilar 2004).

This article does not pursue to provide a biography of Carlos Asensio. Yet, it is useful to outline some aspects of his earlier life that shaped his ensuing professional itinerary. The son of a pharmacist, Asensio was born in Oviedo in 1925, studied pharmacy in Madrid, and for several years alternated his occupation between the family dispensary and completing a thesis on clinical biochemistry at the Casa de Salud de Valdecilla in Santander, entitled *Biochemical Studies on Phosphatases in Relation to Human Clinic*, supervised by Enrique Cavayé Hazen, which he disserted at the Complutense University of Madrid in 1954. Following the advice of S. Ochoa, Asensio then joined the Biological Research Centre (acronym CIB in Spanish) of the CSIC as a grant holder under A. Sols during 1956–1957. This association was critical in Asensio’s scientific career, establishing a fruitful lifelong interplay between the two scientists. In 1959, again on Sols’ advice, he went to B. H. Horecker’s laboratory at the University College of Medicine in New York (nearby Ochoa’s Department of Biochemistry). Asensio worked there until 1961 as a young biochemist and analytical enzymologist on the transport of galactose and the biochemistry of the enzyme galactose oxidase. But his years the USA also endowed Asensio with two key perspectives that shaped his future undertakings. First, working in the international atmosphere of the New York Department made him understand the universality of all scientific knowledge. This sense grew to a new level when—after his stay in Horecker’s Group—the Rockefeller Foundation invited him to create and lead the Department of Biochemistry at the Faculty of Medicine of San Salvador, where he worked for almost 2 years in the very different cultural and economic landscape of a developing country. The second eye-opener he experienced in the USA was elicited by his participation in the summer of 1960 in the legendary *General Microbiology Course* directed by Cornelius van Niel from 1930–1962 at the Pacific Grove Maritime Station in Monterey Bay, California. This course not only led him to view microbiology and biochemistry within the context of the *logique du vivant* (logic of life*)* as François Jacob described a few years later (Jacob 1970) but also to discover ecology as the frame where every biological phenomenon needs to be interpreted. It is noteworthy that many of his whereabouts during Asensio’s time in the USA and El Salvador were written down in a lively and informal style in a continuous flow of correspondence with Sols and other members of his earlier Enzymology Department in Madrid (Sols 1998). Finally, Asensio returned to Spain at the end of 1963, joining the Department of Enzymology of the CSIC, directed by his long-time mentor, A. Sols. This department, which evolved to become the Institute of Enzymology and Molecular Pathology, became part of the Faculty of Medicine at the Autonomous University of Madrid, being one of the first mixed centres between the University and CSIC. This fusion between the University and the Research Council, later replicated elsewhere, was decisive in creating a vibrant cross-fertilisation, as he liked to call it, from which both partner institutions benefited.

While still focused on enzymology for quite a few years upon his return to Madrid, Asensio recovered one decade later an appetite for microbial ecology which, as mentioned above, was decisively elicited first by his participation on van Niels’ Course and quite a few years after by his meeting F. Baquero on occasion of the latter’s Thesis in 1973. Following the first reports on microcins (see above), they both organised in a doctoral course entitled *Microbial Ecology: Biochemical and Genetic Bases* at the Faculty of Medicine of the Autonomous University of Madrid. This course and the many discussions it catalysed sparked a deeper insight into the epistemology of scientific knowledge, as embodied in the process that led to the discovery of such new class of bioactive molecules.

## The there must be principle

A notable conceptual consequence drawn from Asensio’s creative activity is the value of intuition in the process of understanding. This is what we could call the principle of *there must be*. Many scientific discoveries have not arisen from linear reasoning but from explanatory intuition (Seligman and Kahana 2009). It might well be that the *there must be* principle is probably a fusion of logical thinking and intuition (Baquero and Moya 2012). We have examples of this, such as the discovery of the Higgs boson, a subatomic particle hypothesised for decades to explain the current cosmological theory but only found in 2012 (Arbey et al. 2012). Another classic example is, in astronomy, the discovery of the planet Neptunus, which was mathematically predicted but not observed until Johann Gottfried Galle found it with a telescope, exactly where it had been predicted from Le Verrier’s calculations (Airy 1847). In biology, the *there must be* principle has allowed for the greatest advance in molecular biology in the twentieth century by postulating that there had to be a mechanism that allowed the decoding of the DNA message and subsequently translating it into proteins. This approach led to the crucial discovery of messenger RNA (Jacob and Monod 1961; Gros 1980).

Epistemologically, *there must be* implies a kind of *anticipation* of where and how to look for what we are after. In the case of microcins, Asensio became interested in the phenomena of microbial successions occurring during the normal colonisation process of the intestinal tract in newborns. In this context,* there must be* some molecular mediators eliciting such replacements, but their action *must have* characteristics compatible with other circumstances operating in the intestine, i.e. they *had to be* resistant to degradation by intestinal proteases—as they indeed turned out to be. These notions led to the concept coined by Asensio (1976) of *molecular ecology* as the whole of intermolecular interactions with significance in the intestinal microbial ecosystem.

## Reaching out a wider audience

Instead of keeping the above conceptual frames to the minority of those directly involved in microcins research, it is fair to highlight Asensio’s effort to propagate rigorous scientific thinking among new generations. From 1972 onwards, Asensio and Sols, annually organised a course for biomedical PhD students entitled *The Scientific Method in Biomedical Sciences,* regularly taught at the Faculty of Medicine of the Autonomous University of Madrid. The influence of this course on successive waves of young scientists who attended it and the critical thinking skills acquired was immense. In Spain at that time, with limited intellectual muscle and a significant gap between sciences and humanities, PhD students in this course were confronted with Popper’s falsifiability, Kuhn’s paradigm shifts, and Monod’s relational logic. Special mention must be made of the enormous influence that the works of Jacob and Monod had on Asensio’s approach to the scientific creation process, particularly their research on the regulation of the *lac* operon and the discovery of allosteric regulation of certain enzymes. In particular, Asensio’s angle was making sense of biochemical and microbiological results in the context of the whole, living organism. To his end, he adopted and nurtured Monod’s concept of *teleonomy*, i.e. adopting *purpose* not as a metaphysical choice, but as a hermeneutic frame to make sense of the relational logic of biological (Monod 2014). Students learnt how knowledge is generated through the hypothetical-deductive method that characterises Western science. The theoretical classes of the course were interspersed with practical sessions on presentation preparation (storytelling), seminars, and communication skills. This training was complemented by working seminars delivered by the best local biomedical researchers of the time. Finally, everybody was expected to read and publicly discuss books such as *Advice for a young investigator* (Cajal 2004) and *Recollections of my Life* (Cajal 1989) by the famous Spanish neurobiologist. Further obligatory readings included books by Jacob (1970), Monod (2014), and Kuhn (1997). The concept of *General Microbiology* which was at the core of van Niel’s Course in Pacific Grove was also addressed upon reading of the classical book, *The Microbial World* (Stanier et al. 1970). These courses were truly innovative in the academic panorama of the time. Moreover, the program was intended to be linked in real time with participants’ research practice. This inevitably led to lasting learning of the conceptual scaffold on which modern biosciences are based. Unfortunately, the course ceased to be taught after Asensio’s passing and has never been reissued in the same format. Nevertheless, its influence on the dozens of young researchers who passed through it over the years has permeated the work of several generations of microbiologists and biochemists who followed.

## A plea for rigorous—yet elegant—research

**Fig. 6:**
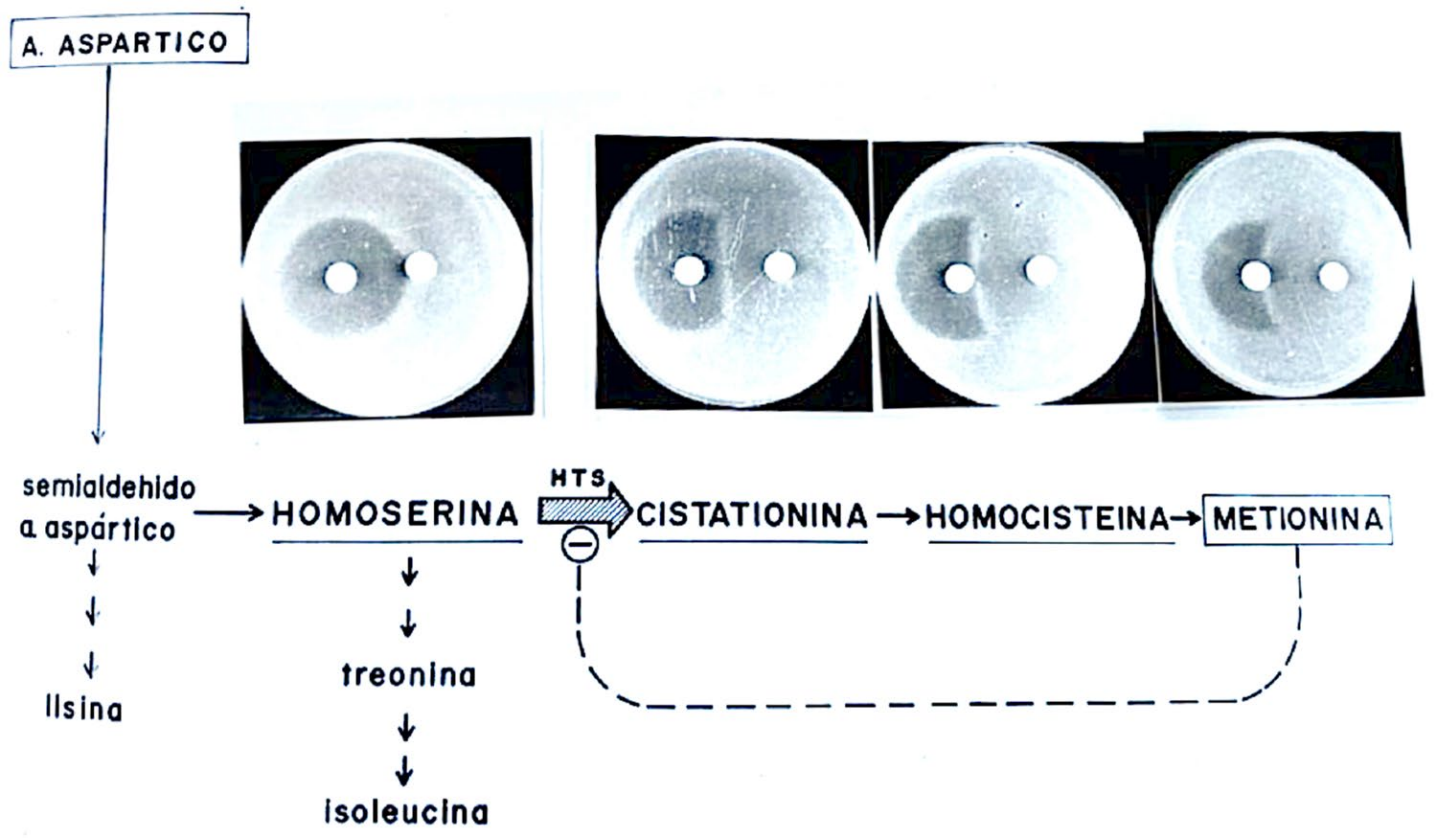
Determination of the mechanism of action of microcin 15 m. Biosynthetic scheme of the amino acid methionine showing inhibition of the enzyme homoserine-*O*-transsuccinylase (HTS), the first specific enzyme of the methionine biosynthetic pathway (image provided by A. Aguilar) by microcin 15 m. This compounds acts on HTS as a false end-product of the methionine pathway, reversibly inhibiting its synthesis and thus inhibiting bacterial growth. This inhibitory action could be antagonised by the metabolites cystathionine, homocysteine, and methionine itself, but not by homoserine (Aguilar 1979; Aguilar et al. 1982b, a). The image reproduces a poster which was hung from the wall of the Laboratory B19 in the Instituto de Enzimología where the work mas made (photo kindly provided by A. Aguilar from his laboratory notes in Spanish)

Throughout his career, Asensio maintained a manifest ability to combine rigorous scientific inquiry with an aesthetic finesse that was evident in the design of his experiments. In this sense, Asensio embodied a type of researcher capable of unapologetically merging scientific creativity with artistic sensitivity. Indeed, throughout his life as a researcher, he never abandoned his interest in architecture, which he once considered as a profession before deciding on Biochemistry (Sols 1998). This should come as no surprise because architecture, like no other discipline, merges technical severity with aesthetic expression. It is interesting that in Spain in the 1970s, talking about combining science with art would have been subject to jokes and ridicule in academic circles. However, the aesthetic and structural vision accompanied Asensio throughout his life and imbued his research activities. In this regard, experiments should be elegant rather than brute-force driven. Therefore, it was disheartening for Asensio that after the discovery of microcins through a simple and elegant experiment (Fig. 3), subsequent attempts to purify these molecules were fundamentally based on brute force. Only years later, with very pure preparations of the compounds in question, were experiments not only highly informative but also highly aesthetic from an intellectual and visual standpoint. One of the best examples of this was the elucidation of the mechanism of action of the so-called microcin 15 m as a reversible inhibitor of the enzyme homoserine trans-succinylase (Aguilar 1979; Aguilar et al. 1982b, a) as shown in Fig. 6. The tension between elegant experiments and those generated through brute force returns to the present day due to the growing abandonment of the hypothetic-deductive method that has dominated molecular biology in recent decades, in favour of exclusively data generation and its computational processing by automated learning platforms. Nevertheless, the scientist must envisage science in their mind, a task that is both logical and artistic (Baquero and Baquero-Braun 2023).

## Inserting science in the societal agenda

Apart from research contributions and conceptual developments, there are other aspects that tend to go unnoticed but form part of Asensio’s contributions to the generational landscape of the time, entirely framed within the political transition undergone by Spain from 1974 to 1982. Immediately after the discovery of microcins, Asensio foresaw a real possibility of exploiting them as new antibiotics at a time when resistance to conventional antimicrobials was not yet the problem we face today. But a continuous search for new antimicrobials was already of great interest. Based on this, one of the first major academia-industry collaborative projects was organised through the formation of a consortium between public institutions: CSIC, Ramón y Cajal Hospital (where Baquero moved to in 1977) and pharmaceutical companies Antibióticos SA and Laboratorios Normón España. This consortium was partly financed by the Concerted Plan 3/76 of CAICYT (Advisory Commission for Scientific and Technical Research) and partly by the Health Research Fund of the Ministry of Health and Social Security. Such public–private funding arrangements, quite common today, were not only exceptional at that time but also viewed with some scepticism from sectors of both the academic and industrial worlds.

Beyond his pioneering role in fostering academia-industry collaborations in Spain, Asensio’s ground breaking efforts in integrating science with the political agenda deserve special recognition. During the period under review, concepts such as innovation, development, technological hubs, university-industry collaboration, and scientific policy were non-existent. In the 1960s and early 1970s, under a dictatorship regime, there was a cautious and controlled opening, with a clear focus on developmentalism in healthcare (through the establishment of large public health hospitals), tourism, and industry. The academic sector, including universities and the CSIC, also experienced significant growth during this time, marked by the creation of new universities and CSIC institutes. The return of young scientists, educated in the USA, Germany, Great Britain, and other leading nations, brought a new notion of excellence. Upon their return, these scientists assumed prominent positions in public institutions, particularly within the CSIC, and significantly transformed the domestic landscape. As part of this generation, Asensio went further to develop, within the Spanish framework, the instruments to integrate scientific research with innovation and technological and economic development. For this purpose, it was necessary for the country to adopt a scientific policy, non-existent in those years. Also, in line with the discovery of microcins, Asensio argued in a notable article, “…*the fruitful interrelationship between scientific discovery and its applied, economic and social projection..*” (Asensio 1977), as a condition for Spain to fully join the developed world. These reflections can be considered precursors to what is now called the *Knowledge-Based Bioeconomy* (KBBE), a concept initially developed in the European Union which in turn inspired many other similar initiatives around the globe (Patermann and Aguilar 2018, 2021; Kircher et al. 2022).

Because of this interest in contextualising science, Asensio had two brief forays into Spanish politics of the time. Firstly, in 1976, he was appointed Deputy Director General for the Promotion of Research, and shortly thereafter as Deputy Director General for Scientific Relations, both at the Ministry of Education and Science. Although Asensio’s political career was brief, during his tenure, some of the foundations were laid for the promulgation of the first Science Law in 1986.

## Conclusion

The decade from 1972 to 1982 was a formative period for many of the major themes and principles that shaped contemporary Spanish society, including the role of science in academia, healthcare, and socioeconomics. Despite persistent barriers that still hinder the country’s potential in this area, the Spanish research community is now fully integrated as a significant player in the European and global contexts. However, this process did not happen overnight, and it is fair to look back in time to identify those who, with their pioneering work, catalysed the evolution towards the reality we enjoy today. Among the prominent figures of the era who made a difference stands Carlos Asensio in a prominent position. This man managed to turn being in the right places at the right times into a special legacy, leaving his mark on virtually all the activities he participated in and the people he interacted with. While it is challenging today to find traces of his work beyond his articles in scientific journals and recognition from those who knew him, it is undeniable that his fundamental contributions to the life sciences in our country are perfectly comparable to those of more recognised figures, such as A. Sols, J. Rodríguez-Villanueva, or M. Salas. Asensio’s pioneering research with microcins, now a classic theme in contemporary microbiology (Baquero et al. 2019, 2024), his dedication to training young researchers, his perspective of science as an economic driver, and his artistic sensitivity make him a role model for the new generations of Spanish scientists, contrasting with the traditional view of science developed in an ivory tower. Unfortunately, his premature death in an accident in 1982 cut short a career that was still in its growth phase, but he left a legacy that deserves to be acknowledged, remembered, and celebrated. Spanish scientists, particularly microbiologists and biochemists, owe him a debt of gratitude.

## Data Availability

No datasets were generated or analyzed during the current study.
